# The Effects of Heat Exposure on Human Mortality Throughout the United States

**DOI:** 10.1029/2019GH000234

**Published:** 2020-04-01

**Authors:** Drew Shindell, Yuqiang Zhang, Melissa Scott, Muye Ru, Krista Stark, Kristie L. Ebi

**Affiliations:** ^1^ Nicholas School of the Environment Duke University Durham NC USA; ^2^ Duke Global Health Initiative Duke University Durham NC USA; ^3^ Porter School of the Environment and Earth Sciences Tel Aviv University Tel Aviv Israel; ^4^ Now at the Samuel DuBois Cook Center on Social Equity Duke University Durham NC USA; ^5^ Center for Health and the Global Environment University of Washington Seattle WA USA

**Keywords:** heat‐related mortality, climate change, heat exposure

## Abstract

Exposure to high ambient temperatures is an important cause of avoidable, premature death that may become more prevalent under climate change. Though extensive epidemiological data are available in the United States, they are largely limited to select large cities, and hence, most projections estimate the potential impact of future warming on a subset of the U.S. population. Here we utilize evaluations of the relative risk of premature death associated with temperature in 10 U.S. cities spanning a wide range of climate conditions to develop a generalized risk function. We first evaluate the performance of this generalized function, which introduces substantial biases at the individual city level but performs well at the large scale. We then apply this function to estimate the impacts of projected climate change on heat‐related nationwide U.S. deaths under a range of scenarios. During the current decade, there are 12,000 (95% confidence interval 7,400–16,500) premature deaths annually in the contiguous United States, much larger than most estimates based on totals for select individual cities. These values increase by 97,000 (60,000–134,000) under the high‐warming Representative Concentration Pathway (RCP) 8.5 scenario and by 36,000 (22,000–50,000) under the moderate RCP4.5 scenario by 2100, whereas they remain statistically unchanged under the aggressive mitigation scenario RCP2.6. These results include estimates of adaptation that reduce impacts by ~40–45% as well as population increases that roughly offset adaptation. The results suggest that the degree of climate change mitigation will have important health impacts on Americans.

## Key Points

- We develop a generalized risk function from U.S. epidemiological data to quantify nationwide heat‐related premature deaths
- We find ~12,000 premature deaths annually in the contiguous United States during the 2010s
- Projected deaths rise to ~110,000 and ~50,000 year^−1^ under high‐ and moderate‐warming scenarios, respectively, including population growth

## 1 Introduction

Climate change is altering the characteristics of multiple hazards that can adversely affect human health, including the spread of vector‐borne and water‐borne diseases, undernutrition and food safety and security‐related illnesses, and exposure to weather extremes such as heatwaves, floods, and storms. We focus on the current impacts and projected risks of changing exposure to high ambient temperatures. Exposure to heat can compromise the body's ability to regulate its internal temperature, potentially resulting in heat exhaustion, hyperthermia, worsening of chronic conditions, and heatstroke, leading to temperature‐related deaths (Sarofim et al., 2016). Temperature and mortality are linked not only at hot extremes, such as during heatwaves, but also at temperatures that are moderately hot (Gasparrini et al., 2015; Lee et al., 2014). Owing to their more frequent occurrence, small temperature changes at mild or moderate temperatures can have larger health impacts than changes at extreme levels, such as during heat or cold waves (Gasparrini et al., 2015; Sarofim et al., 2016; Wellenius et al., 2017). We do not address cold‐related mortality given its complexities and uncertainties. In particular, it is unclear to what extent empirically derived cold‐related impacts are likely to reflect causal mechanisms given that other confounding effects such as influenza occur during winter seasons.

U.S. temperature‐mortality studies have documented the health consequences of exposure to hot temperatures and heatwaves, typically examining groups of cities (Anderson & Bell, 2009; Gasparrini et al., 2015; Honda et al., 2014; Lee et al., 2014; Schwartz et al., 2015; Weinberger et al., 2017). One national‐level study statistically related annual average U.S. death rates to the frequency of daily temperatures in ten 10 °F bins (along with precipitation; Deschenes & Greenstone, 2011). To provide a full geographic analysis of the temperature‐mortality relationship across the contiguous United States, we utilize observed risk relationships based on epidemiological studies in multiple U.S. cities, as in prior work, and then develop a generalized risk function to capture the geographic variation in the observed functions by including a dependence upon local climatological conditions. We evaluate the ability of the generalized function to reproduce results obtained using the observed risk functions, then apply it to analyses of the health risks of projected climate change in the United States under several scenarios. We include projected changes in population and perform analyses both with and without accounting for potential adaptation.

## 2 Methods

Calculation of premature mortalities due to heat exposure is based upon the exposed population, their baseline mortality rate, and the increased risk associated with exposure to a given temperature level at a particular location. We describe the methods used to evaluate each of these factors in this section.

### 2.1 Population and Baseline Mortality Rates

We use 0.5° × 0.5° population data for 2010 from the Gridded Population of the World version 4 (CIESIN/FAO/CIAT, 2017). As our calculations are based on daily heat exposure, we require estimates of daily baseline mortality rates. We base these on annual all‐cause mortality for the United States in 2010 from the Global Burden of Disease database (Global Burden of Disease Collaborative Network, 2018), weighted by monthly mortality rates observed in Northern Hemisphere countries from 2000 to 2010 (Marti‐Soler et al., 2014). The weighting assumes that each month's deaths are equally distributed within the month, as in prior studies (Schwartz et al., 2015).

### 2.2 Exposure‐Response Function

**Figure 1:**
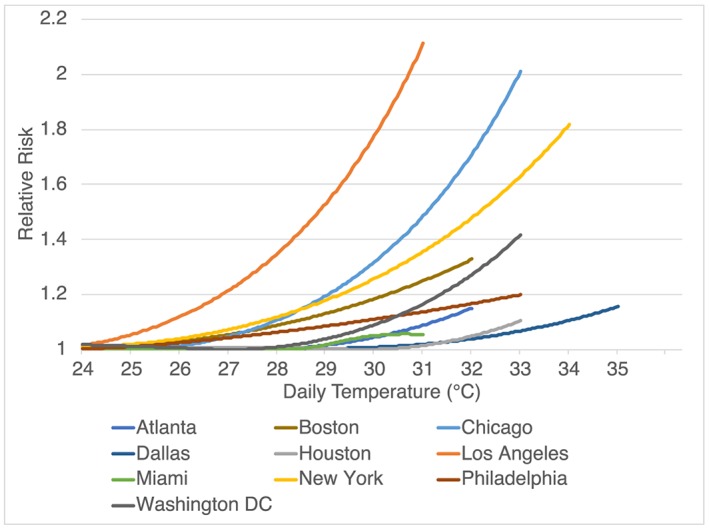
Relative risk functions for all‐cause all‐age mortality as a function of daily temperature for 10 U.S. cities as documented in Weinberger et al. (2017). Summer mean temperatures for these cities are Atlanta 25.7°C, Boston 21.2°C, Chicago 22.5°C, Dallas 28.9°C, Houston 28.4°C, Los Angeles 21.8°C, Miami 28.4°C, New York City 23.1°C, Philadelphia 23.9°C, and Washington DC 24.4°C.

We begin with observed exposure‐response functions (ERFs) for the associations between mean daily temperature from the National Oceanographic and Atmospheric Administration (NOAA) and daily all‐cause all‐ages mortality from the National Center for Health Statistics for 10 cities over 1985–2006. These were derived using distributed lag nonlinear models with a 21‐day lag function to obtain initial ERFs from which the best linear unbiased prediction was then calculated using a meta‐analytic model (Weinberger et al., 2017). The cities are Atlanta, Boston, Chicago, Dallas, Houston, Los Angeles, Miami, New York, Philadelphia, and Washington D.C. (Figure 1). The ERF was calculated as relative risk (RR) per tenth of a degree increment of temperature for each city, with observed daily mean temperatures ranging from −26 to 35°C. The RR is defined to be 1 at the temperature at which minimum mortality was observed for each city (hereafter referred to as the optimal temperature [OT]).

We fit second‐order polynomials to the individual cities' raw data with the equation RR = 1 + *aT* ^2^ + *bT*, where *T* is daily temperature in °C above OT (no constant term is included in the polynomial so that RR will be one when *T* equals OT). Observing that the shape of the ERF appeared to vary according to the climatology of the cities, with generally steeper curves in colder places (Figure 1), we hypothesized that a generalized function might be able to realistically capture observed geographic variations if it were related to summer mean temperatures (SMT; June–August). We therefore ran linear regressions between the coefficients *a* and *b* against city‐specific SMT averaged across 1985–2006, again using temperatures from NOAA (Office of Oceanic and Atmospheric Research/Earth System Research Laboratory Physical Sciences Division, Boulder, Colorado, USA; https://www.esrl.noaa.gov/psd/). We then used the slope and intercept of the linear fits to create a generalized risk function for hot temperatures above OT:

(1) $$RR=1+a_{s}\times \left(\text{SMT},-,a_{i}\right)T^{2}+b_{s}\times \left(\text{SMT},-,b_{i}\right)T$$

where *a*_*s*_ and *b*_*s*_ are the slopes of the respective coefficient fits vs. SMT, *a*_*i*_ and *b*_*i*_ are the *x*‐intercepts of the respective coefficient fits, and *T* is the daily temperature in °C above OT. To generalize this beyond the 10 cities, we assume that the local OT across the United States is well represented by each location's 84th percentile temperature (Gasparrini et al., 2015; Honda et al., 2014). Inserting the coefficient values from the regression, we obtain

(2) $$RR=1–0.0014\times \left(\text{SMT},-,30.9\right)T^{2}+0.005\times \left(\text{SMT},-,26.7\right)T$$

Note that since SMT is less than 30.9°C in most of the United States, the coefficient of the quadratic term, −0.0014 × (SMT − 30.9), increases as SMT decreases, consistent with the observation that there is generally a stronger increase in RR at the highest temperatures in cooler locations. The quadratic and linear terms are of opposite sign so that this equation might behave unrealistically if used outside the range of SMT seen in the contiguous United States (roughly 20–30°C). For SMT less than 26.7°C, this equation can lead to RR < 1 for small values of *T*, and hence, we impose a lower limit on the RR of 1. At SMT values greater than 30.9°C, RR would decrease with increasing *T* for large values of T (i.e., increasing temperatures would reduce risk). Such behavior, though possible (e.g., the Miami curve in Figure 1), would require further study to confirm, but such conditions are almost never (<0.1%) encountered in our analysis. Finally, we note that an alternate form of the equation, RR = 1–0.0011 × (SMT − 32.3)*T* ^2^ + 0.0032 × (SMT − 26.6)*T*, is obtained if each of the individual 10‐city polynomial fits is forced to go through RR = 1 at *T* = 0 rather than finding the best fits while accepting a slight offset at *T* = 0. This method yields slightly poorer fits to the observed ERFs for the individual cities but is arguably as sensible a procedure and produces a very similar generalized equation. Results are therefore insensitive to this choice, however, with differences generally of a few percent and far smaller than other sources of uncertainty.

**Figure 2:**
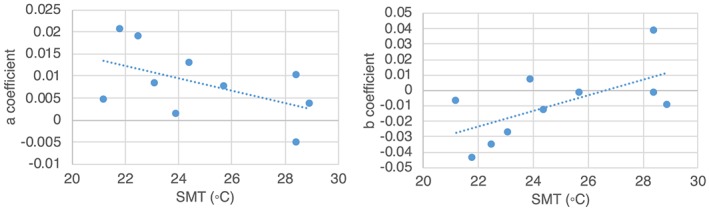
Coefficient values for the exposure‐response function binomial fits for each of the 10 cities and the regression across those points. *R* values for the regression are (a) 0.51 and (b) 0.62.

The 10 ERFs used here are a relatively small sample size, leading to fairly weak correlations for our coefficient fits (Figure 2) and a substantial sensitivity to individual data points. Examining the values of *a*_*s*_ and *b*_*s*_ calculated leaving out data from one city at a time, we find that *a*_*s*_ and *b*_*s*_ values vary by up to ~40%, though the standard deviation across the 10 results is 20–30%. Hence, the generalization procedure would likely benefit from additional data, and results for individual cities will be difficult to characterize accurately (as discussed further in section 3).

### 2.3 Heat‐Related Premature Deaths

Heat‐related deaths are then calculated as the product of daily baseline mortality times population times (RR − 1)/1, where the latter term is referred to as the attributable fraction. Daily temperatures used in calculating RR are based on simulations driven by the Representative Concentration Pathways (RCPs) with year 2100 radiative forcing targets of 2.6, 4.5, and 8.5 W/m^2^, using results from the database developed by the Inter‐Sectoral Impact Model Intercomparison Project (ISIMIP2b, https://www.isimip.org/, accessed 9 January 2019; Warszawski et al., 2014). The ISIMIP2b used observational data to perform bias corrections (Frieler et al., 2017). We utilize data from five bias‐corrected general circulation models from the Coupled Model Intercomparison Project Phase 5 (CMIP5): HADGEM2‐ES, GFDL‐ESM 2M, IPSL‐CM5A‐L, MIROC‐ESM‐CHEM, and NorESM1‐M These five GCMs were shown to be representative of the range of projections of future climate across the CMIP5 models (Warszawski et al., 2014). The model outputs were downscaled through bilinear interpolation at a 0.5° × 0.5° spatial resolution and linear interpolated by day of the year. Simulations with these models began in 2005 and extended through 2100. We use models for the near past as well as for the future to ensure consistency across time periods and to avoid potential problems with missing values in observational datasets. We report results as mean annual averages over the years examined.

Uncertainties arise from the ERF and temperatures (and in our case from the generalization of the ERF, discussed in section 3). Uncertainty ranges for individual cities can be extremely large, with 95% confidence intervals (CIs) varying by up to −121 to +132% for cities with at least 100 deaths over 1992–2002 (Weinberger et al., 2017). Values summed across the 10 cities show much narrower CIs, however, assuming no correlation in error across cities (as the ERF and temperature uncertainties are unlikely to be largely systematic). In the case of sums, the uncertainty range is ±35% averaged across the reported total for the recent past, 2050s and 2090s (Weinberger et al., 2017). As we emphasize large‐scale results in this work, we utilize this uncertainty range associated with the 10‐city sums in our analyses.

## 3 Evaluation

Comparison of the ERF for each of the 10 cities shows that the generalized function is able to capture the observed shape of the curve very well. The values of the *R* ^2^ correlations between these curves range from 0.96 to 1.00 over 9 of the 10 cities. The only city with a lower value is Miami, where the correlation is 0.88. Unlike the ERF for other cities, risks in Miami decrease with increasing temperature near the highest recorded temperatures (Figure 1; Miami is the ERF ending at 31°C). This accounts for the lower correlation, as our second‐order polynomial does not capture that shape over Miami's temperature range. However, that response is both an outlier across cities and is well within the uncertainty of the ERF at the highest temperatures, as that uncertainty grows rapidly at the highest values since they occur relatively infrequently (e.g., relatively uncertainty is ~15% at half a degree below maximum, while it is ~35% at the maximum value). Hence, we believe that it is most appropriate to retain the second‐order shape across all locations.

Having established that the generalized function can broadly capture the observed local ERFs, we next evaluate the results using temperatures in the earliest period of the model scenarios, 2006–2010 (Table 1). This is a stricter test, as two similarly shaped polynomials can have a high correlation even when their magnitudes differ substantially. In the five cities for which use of the observed ERF yields at least 100 deaths, the results using the generalized ERF are low by ~20–66% with the exception of New York for which there is a 38% overestimate. For the five cities with fewer than 100 heat‐related deaths, absolute biases are likely more useful, and these range from −11 to +69. As differences between results using the generalized and observed ERFs are systematic, this comparison is largely insensitive to the years including in this analysis (i.e., the relative/absolute error values are within a few percent/persons using any four of the five sampled years for cities with more/less than 100 deaths). Hence, overall, biases at individual cities are typically substantial in either the absolute or relative sense. There is no obvious systematic bias in cold or warm cities. For example, the generalized ERF overpredicts deaths in cooler cities such as New York, Philadelphia, and Boston but underpredicts in Chicago and Los Angeles, which are also cool. Similarly, deaths are slightly too low in hotter cities such as Atlanta or Miami but too high in Dallas, which is also hot. Consistent with errors being predominantly randomly distributed, the total across the 10 cities using the generalized ERF is very close to the total using each individual city's observed ERF. In addition, the reported observed ERFs have a relatively large uncertainty range themselves (Table 1). In fact, for all but two of the cities (Los Angeles and Dallas), the bias introduced by our use of a generalized equation is within the uncertainty range reported for an individual city. For Dallas, the bias range across years does overlap with the reported uncertainty range, and for Los Angeles, the bias is essentially the same magnitude as the reported uncertainty range. We hence use the average difference in the total (7%) as part of our characterization of the uncertainty due to the use of the generalized equation when presenting U.S. totals, though we caution that values for individual locations would have much larger uncertainties using our generalized method.

**Table 1:** Annual Average Deaths Attributable to Heat Exposure Averaged Over 2006–2010 Using Generalized and Observed ERFs

City	Generalized function	Observed function	Absolute error	Relative error (range)	Observed relative low error (%)	Observed relative high error (%)
Atlanta	83	105	−23	−22% (−18, −58)	−255	400
Boston	149	83	66	79% (67, 84)	−228	241
Chicago	290	503	−212	−42% (−38, −80)	−63	107
Dallas	86	17	69	419% (188, 672)	−377	368
Houston	32	35	−3	−9% (63, −3)	−533	767
Los Angeles	61	182	−121	−66% (−62, −79)	−57	64
Miami	32	43	−11	−25% (−15, −36)	−269	300
New York	507	368	139	38% (13, 49)	−81	86
Philadelphia	92	46	46	99% (32, 119)	−121	132
Washington D.C.	114	177	−63	−36% (−35, −40)	−135	295
Total	1,446	1,559	−113	−7% (−3, −17)	−41	43

*Note*. The range for relative error is across the years examined.

Abbreviation: ERFs: exposure‐response functions.

**Figure 3:**
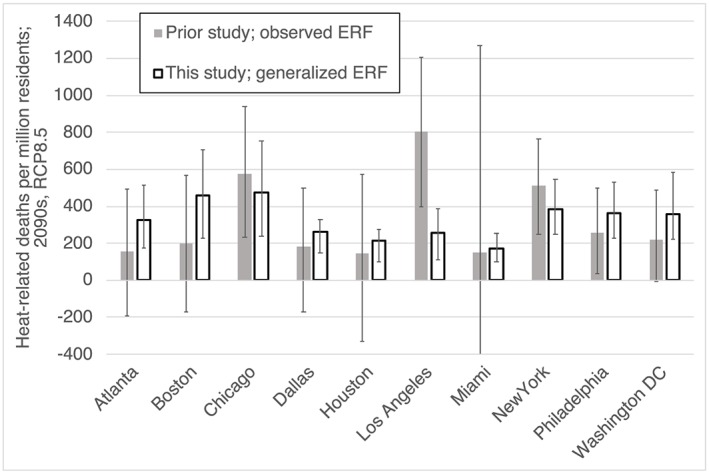
Heat‐related deaths for the indicated cities near the end of the century under Representative Concentration Pathway (RCP) 8.5 without population changes or adaptation. Values from Weinberger et al. (2017; filled bars) are annual averages for 2085–2095, whereas values from this study (open bars) are for 2090–2099, normalized to match county‐level population used in the earlier work. Uncertainties for Weinberger et al. (2017) include exposure‐response function (ERF) and temperature projections (and extend off the chart to −1,500 for Miami), whereas for this evaluation of the generalized equation we show ranges across the five models used for temperature projections.

Finally, to further evaluate our generalized equation, we examined the effects of the warmer U.S. climate projected under RCP8.5 in 2100 in comparison with prior results. Our results are largely consistent with those from an earlier study using observed ERFs (Figure 3). Despite a markedly lower mean estimate for Los Angeles and substantially higher values for Atlanta, Washington D.C., and Boston, our mean values are within the uncertainty range from that study in all cities except Los Angeles. Including the variation in our projected temperatures across the different climate models, our values overlap the central value of the previously reported ranges in seven cities and the full range in nine cities. As with the recent past, biases appear randomly distributed. Our total across the 10 cities for 2100 is 20,655 (95% CI 12,784–28,526; accounting for the full uncertainty in our calculation including generalization bias and normalizing for population differences to facilitate comparison), which is in good agreement with the value of 26,237 (95% CI 17, 673–33,961) reported by Weinberger et al. (2017). If anything, our method seems conservative, yielding total values that are ~21% less than those using observed ERFs in this evaluation.

Overall, we demonstrated that although our generalized ERFs lead to substantial uncertainties at the city level, they introduce relatively minor biases at the larger scale, producing values within ~10–20% of the sum of values using observed individual city ERFs. To characterize the uncertainty introduced by our use of generalized ERFs at the large scale, we take the average of the absolute biases in our evaluation of recent‐past and projected future impacts, obtaining a value of 14%. This bias is markedly less than uncertainty range for summed values of ~35% described previously accounting for uncertainties in epidemiologically derived ERFs and projected temperature changes. Thus, we are confident that calculations using our generalized ERFs can provide valuable insight into changing nationwide U.S. health risks of heat exposures due to climate change. As these are assumed to be independent sources of error, we sum these in quadrature to derive the total uncertainty range (38%), which we use hereafter for nationwide analyses.

## 4 Nationwide Heat‐Related Deaths

### 4.1 National Totals

We apply our generalized ERFs to the entire contiguous United States, comparing results for the last decade of simulations (2090s; 2090–2099) for the three RCPs against the first full decade of those simulations (2010s; 2010–2019). We include projected changes in population based on country‐level data for a United Nations' medium fertility scenario (United Nations, 2011). It has long been recognized that adverse impacts occur at hotter absolute temperatures in hot areas than in cool ones in the United States (e.g., Curriero et al., 2002), suggesting that people are in some way adapted to their climatological conditions. Accounting for adaptation is complex, however (e.g., Wang et al., 2018), as it is difficult to separate socio‐economic changes from physiological adaptation in analysis of historical data and difficult to project socio‐economic trends. We therefore include two potential representations of adaptation in our analysis.

In our base case without adaptation, we use the values of OT and SMT for each location from the 2010s in our projections of future risks. In the first adaptation method, we hypothesize that people living in cooler climate develop shallower risk curves as they acclimatize to warmer summers. This is accomplished by using the 2090s SMT values in the future analyses and hence is referred to as “adapting to SMT” (it can be thought of as “Northerners become like Southerners”). This could represent physiological changes or changes in the built environment or personal habits that allow people in cooler climate to reduce impacts of heat exposure (e.g., construction of “cooling centers” for those without air conditioning, better awareness, and knowing what to do when it is hot). In our second method, we instead assume that people adjust to the changing distribution of temperatures rather than the summer climatology, so that what used to be considered “extreme” is no longer so in the future. This is accomplished by using the 2090s OT values in the future analyses (it can be thought of as “the new distribution becomes the new normal”). As extreme daily temperatures are by definition more variable than summer mean values, we assume that people can adjust to half the change in the daily distribution over this century (this is most important for the RCP8.5 case in which warming occurs very rapidly), similar to the “lagged‐adaptation” approach used elsewhere (Anderson et al., 2018). We therefore present the average of the baseline and 100% response to the OT changes for this case and refer to this as “lagged adaptation to OT.”

**Figure 4:**
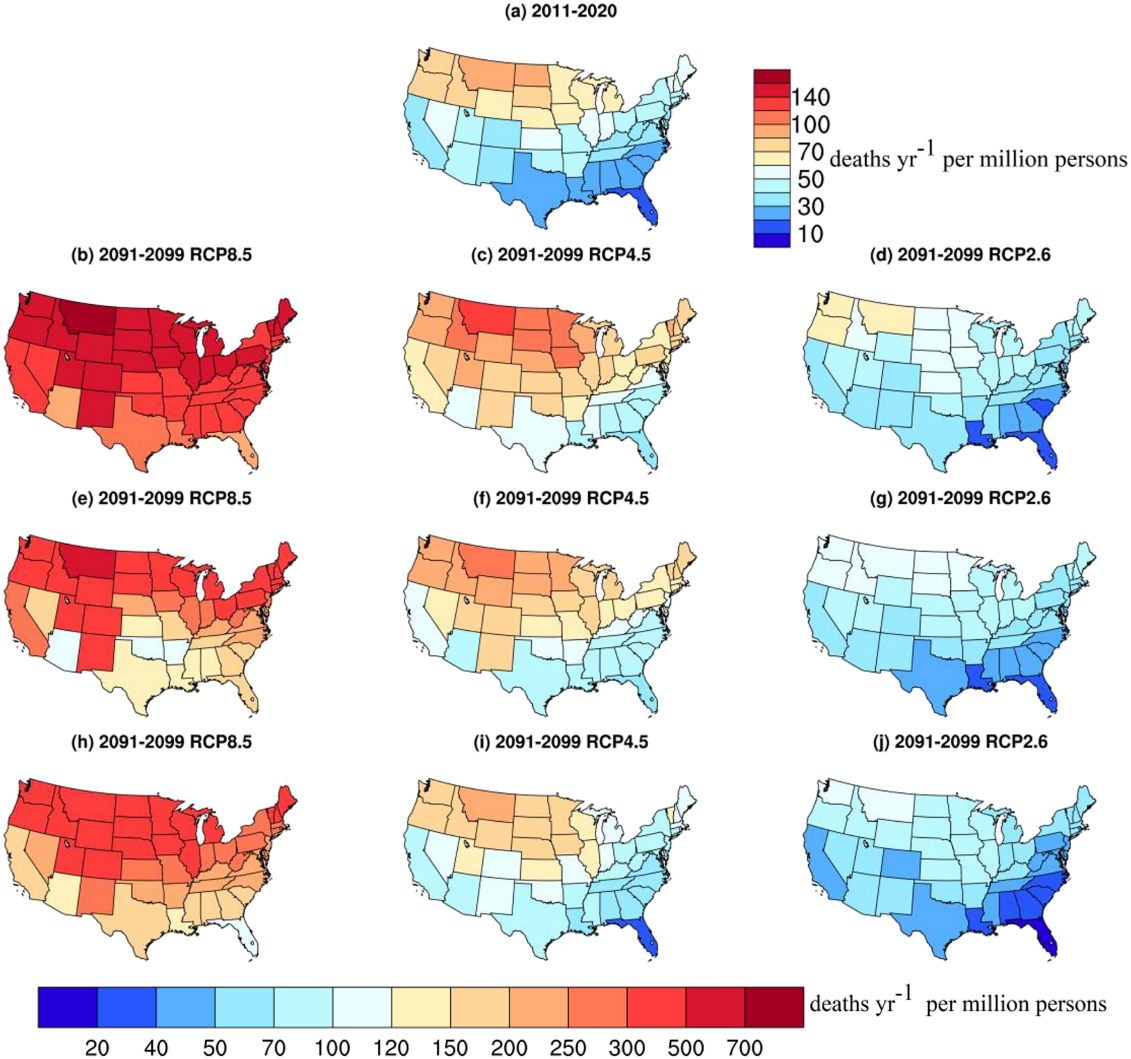
State‐level heat‐related mortality. Values are shown in deaths/year per million persons by state for the indicated years and for the indicated future scenarios including (b–d) no‐adaptation, (e–g) adaptation to summer mean temperatures, or (h–j) adaptation to the change in temperature distribution (“lagged adaptation to OT”). Note the change in scales between near‐present (a) and future (b–j).

During the 2010s, we estimate 12,000 (95% CI 7,400–16,500) annual average premature heat‐related deaths. Per capita heat‐related death rates are greatest in northern states and least in the South (Figure 4). The total is very close to 10 times the 10‐city sum of 1,446, comparable to the ratio of the total U.S. population of ~320 million to the 34 million included in the 10‐city‐centered grid boxes. This is not altogether surprising given that the 10 cities are broadly representative of the SMT range experienced throughout the contiguous United States, but implies that the distribution of daily temperatures in areas outside the 10 cities was also broadly consistent. Unsurprisingly, the results also indicate that sums over a subset of U.S. cities miss a large portion of the total national impacts. Note that across three available distinct sets of model output for the 2010s, multimodel mean U.S. totals were within 3% of the mean, demonstrating that analysis of 10 years of model output is adequate to provide robust results.

**Figure 5:**
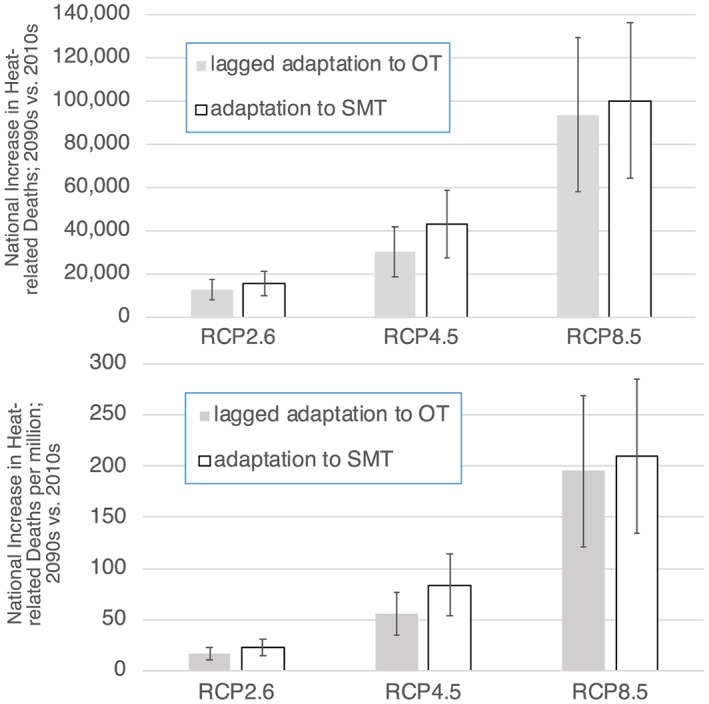
(top) Total and (bottom) per capita increases in nationwide U.S. heat‐related deaths over the 21st century under the three scenarios. Values include projected increases in population and account for potential adaptation.

By the last decade of this century, the total number of U.S. deaths, accounting for adaptation, rises by a factor of about 9 under RCP 8.5 to reach more than 100,000 annually. Under RCP4.5, it increases by a factor of ~4–5, whereas under RCP2.6, national‐level heat‐related deaths roughly double (Figure 5). At one extreme, if we do not account for adaptation at all, the increases are nearly double those seen when accounting for adaptation. This result is largely insensitive to the type of adaptation considered, as the increases in deaths for the 2090s relative to the 2010s under RCP8.5 are 54% and 57% of the no‐adaptation values for lagged adaptation to OT or adaptation to SMT, respectively. At the other extreme, if both types of adaptation are included simultaneously, the deaths increase under RCP8.5 by only 11% of the no‐adaptation value. Comparing across RCPs, the effects of adaptation under RCP4.5 are smaller, so that the rise in 2090s deaths is closer to that seen in the no‐adaptation case with values of 60%, 85%, and 44% for lagged adaptation to OT, adaptation to SMT, or both, respectively. Finally, if we again include adaptation to both SMT and OT but in this case non‐lagged for both factors, increases in deaths are only 5% and 12% of their no‐adaptation values under RCP8.5 and RCP4.5, respectively. Hence, any rise in deaths is nearly eliminated under such an extreme case and is 98–100% eliminated on a per capita basis, although fully synchronous adaptation to all aspects of changing temperatures seems implausible.

There are limited results from other studies against which to compare these results because, as noted, most prior studies were based on epidemiology from a select set of U.S. cities and look at either hot and cold temperatures or only heat extremes. For instance, estimated changes in annual premature death rates for 49 U.S. cities by the U.S. EPA (2017) show regional increases of up to about 100 per million people for 2080–2099 under RCP8.5, whereas we find values of several hundred per million people for the same scenario (Figure 4). Their values, however, include changes in both heat and cold extremes (not just heat), do not include heat that is above OT but not “extreme,” and cover only 49 cities, so our estimate should be higher. These estimates are also based on simulating the effects of the RCP8.5 scenario with a different set of climate models. Perhaps more useful is to compare estimates of the effect of adaptation. U.S. EPA examined the adaptation case for which future responses for all cities were equal to the response of the hottest of the 49 cities today, comparable in some ways to our adaptation to SMT. They found that such adaptation led to approximately a 50% reduction in premature deaths, very similar to our findings. We can also compare the sensitivity of impacts to the future scenario examined. The U.S. EPA study reported impacts a factor of 2.4 times larger under the stronger climate change RCP8.5 scenario than under RCP4.5. Our results are again consistent, with 2.3 times larger impacts under RCP8.5 than RCP4.5 for our comparable adaptation to SMT results.

Looking at heatwaves (extreme and moderate) in 82 U.S. communities for 2061–2080, another study found an increase in exposure to high‐mortality inducing heatwaves from 2 million person‐days per year at present to 46–122 under RCP8.5 without adaptation (Anderson et al., 2018). Although they did not evaluate mortality, this suggests a marked increase over present‐day impacts. We can again compare estimates of adaptation. In their study, the increase was muted to 26–71 million person‐days per year with “lagged adaptation” that assumed adjustment to midcentury temperatures, a very similar decline to that seen in our comparable lagged adaptation to OT case that yielded a 46% reduction in the projection obtained without including adaptation.

A purely statistical study, that is, incorporating no epidemiological estimates of risk, found an increase in nationwide U.S. heat‐related deaths of ~123,000 ± 41,000 for 2070–2099 under the A1F1 scenario, a high‐warming scenario similar to RCP8.5 (Deschenes & Greenstone, 2011). Although the methods were relatively simple, for example, regressing annual health data to number of days with temperatures in particular bins rather than accounting for temporally coincident or near‐coincident impacts of heat as in epidemiological studies, the values are surprisingly consistent with our results (Figure 5). In addition to their differing statistical method, they did not include projected growth in population, changes in baseline mortality, or adaptation, however, so their results cannot be rigorously compared with ours.

Finally, the World Health Organization projected increases in heat‐related deaths in North America under a fairly high‐warming scenario, with totals of 7,300 additional deaths in the 2030s and 16,000 in the 2050s relative to 1961–1990 without adaptation (World Health Organization, 2014). These increases are qualitatively similar to results obtained here, but difficult to compare quantitatively owing to methodological differences. In particular, the World Health Organization study included only persons aged 65 and older, used the same ERF everywhere, and analyzed impacts in different years and over a larger area.

### 4.2 State‐Level Values

Looking at the state‐level results, the two estimates of adaptation (adaptation to SMT and lagged adaptation to OT) yield quite similar results (Figure 4). In both cases, the current spatial distribution of per capita heat‐related premature deaths, with larger values in the upper Midwest through the Pacific Northwest and smallest values in the Southeast, persists across the three future scenarios.

Under the highest warming scenario, RCP8.5, the area of maximum per capita impact with either adaptation estimate extends down through the Mountain West through Utah, Colorado, and Arizona, down to Illinois in the Midwest, and eastward to include Michigan and parts of the Northeast, states that do not stand out at present or in the future under the lowest warming RCP2.6 scenario. The method used to estimate adaptation does affect the pattern mildly, however, with differences for Oklahoma and Arkansas under RCP8.5 and for California and Nevada under RCP2.6, but these are generally fairly small variations. Hence, it appears that the dominant distributional change is that under high‐warming scenarios, the area of maximum per capita impact extends south and east from its current location in the northwestern part of the United States.

## 5 Conclusions

Having developed a generalized method to evaluate the effects of exposure to heat on human health in the United States, we calculate impacts for the near‐present and modeled projections over the 21st century under three scenarios. We find a super‐linear response so that premature deaths in the U.S. increase faster than warming across the scenarios. This suggests that unless adaptation is highly effective (e.g., populations adapting to both OT and SMT), it is less likely that heat risks to the U.S. population under high‐warming scenarios could be managed, a concept widely acknowledged in evaluation of future damages (e.g., Kopp et al., 2012). We also find that under high‐warming scenarios, areas that suffer greatly from heat exposure expand substantially to include parts of the United States that are currently not as susceptible to heat. This finding is largely insensitive to assumptions about future adaptation, at least those explored here.

The use of a generalized approach allows us to analyze the entire contiguous United States, in contrast to nearly all prior studies that have evaluated only specific cities. Note that as our epidemiological data come from metropolitan regions, analysis of rural areas would provide a useful additional evaluation of our generalization. The applicability of such a generalization to other parts of the world is a topic worthy of further investigation. Additionally, we have included deaths related to exposure at all temperatures above the minimum risk level, whereas many studies have focuses only on extreme heat. Hence, our values have yielded substantially larger impacts than those reported in most prior work.

This study did not include socio‐economic development as a factor in adaptation. Increased wealth could allow greater access to air conditioning and health care among the portion of the population that current lacks such services. Indeed, trends in adaptation may have as large or larger impact than trends in climate in some instances (Kinney, 2018). Air conditioning access is strongly correlated with reduced heat‐related mortality (Barreca et al., 2016), but many parts of the United States, especially those with warmer climates, are already near saturation. Hence, the adaptation potential may be greater in colder climates, so that there could be convergence between cool and warm climates in the future. Our adaptation to SMT method does not lead to convergence, as all regions adapt, whereas estimates of adaptation based on setting future responses for all cities equal to the response of the hottest city today (U.S. EPA, 2017) do result in convergence. As discussed previously, these methods yield fairly similar large‐scale results in the cases we examined but could substantially affect the relative response across regions and geographic convergence merits further study. Access would also depend on the distribution of income, as the poor spend a much larger fraction of their income on energy and thus can less afford additional air conditioning. Some economic analyses have attempted to evaluate the potential costs of adaptation, such as increased spending on electricity for cooling or rental of air conditioners (Carleton et al., 2019), which could help inform estimates of income‐dependent adaptation rates. Projections of adaptation and socio‐economic changes might also account for government programs to address heat exposure, such as public cooling stations in certain states.

Our results provide an indication of the potential for warming to lead to large increases in the number of Americans who die prematurely each year due to heat exposure, along with a potential expansion in the area of maximum impacts southward and eastward, even assuming adaptation. This implies that significant efforts are required to increase the resilience of vulnerable populations in order to even maintain current (and unacceptable) levels of heat‐related mortality (Astrom et al., 2017), and that substantial public health benefits would be achieved by increased climate change mitigation.

## Conflict of Interest

The authors declare no conflicts of interest relevant to this study.
